# *Rubi idaei fructus* as a Source of Bioactive Chemical Compounds with an Important Role in Human Health and Comparison of the Antioxidant Potential of Fruits and Juice of Three Repeat-Fruiting *Rubus idaeus* L. Cultivars

**DOI:** 10.3390/metabo13111124

**Published:** 2023-11-02

**Authors:** Mirosława Chwil, Renata Matraszek-Gawron, Mikołaj Kostryco

**Affiliations:** Department of Botany and Plant Physiology, University of Life Sciences in Lublin, 20-950 Lublin, Poland

**Keywords:** common raspberry, primary and secondary metabolites, energy value, fatty acids, amino acids

## Abstract

*Rubi idaei fructus* is a source of nutritionally important bioactive chemical compounds, mainly antioxidants, which strengthen the immune system and can be used in the prophylaxis and adjuvant therapies of many oxidative stress-induced diseases. There are no literature reports presenting a comprehensive comparative analysis of the antioxidant activity and nutritionally relevant metabolites contained in the fruits of repeat-fruiting raspberry cultivars, which are commonly grown in Europe. The aim of this study was to carry out a comparative analysis of the antioxidant potential (Folin–Ciocalteu, DPPH, FRAP), the content of selected primary and secondary metabolites, and the qualitative and quantitative composition of amino acids and fatty acids in the fruits of *R. idaeus* cv. ‘Pokusa’, ‘Polana’, and ‘Polka’. The fruits of the analyzed cultivars have a low caloric value (171–219 kcal/100 g); low content of available carbohydrates (6–6.6%) and total carbohydrates (3.4–4.8%); and high levels of dietary fiber (4.7–5.8%), vitamin C (22.8–27 mg/100 g), anthocyanins (25.1–29.6 mg/100 g), and flavonoids (0.5–2.6 mg/100 g). The fruits were found to contain valuable unsaturated fatty acids (35–60%), especially MUFAs with dominant oleic, elaidic, palmitic, and erucic acids and PUFAs (α-linolenic, eicosapentaenoic, and linoleic acids). MUFAs from the ω-9 group accounted for 12–18%, whereas the content of PUFAs from the ω-3 and ω-6 groups was in the range of 15–23 and 6–21%, respectively. Exogenous amino acids, accounting for 56–62%, were dominated by leucine, phenylalanine, and lysine. The following order of the total polyphenolic content was established in the fresh fruit juice from the analyzed cultivars: ‘Pokusa’ < ‘Polana’ < ‘Polka’. The different antioxidant capacity assays used in the study confirmed the high antioxidant potential of the fruits and fresh juice from the three *R. idaeus* cultivars. This indicates that raspberry fruits can serve as a source of nutrients and can be used as a valuable supplement in a healthy human diet and a raw material in the pharmaceutical and cosmetic industries.

## 1. Introduction

### 1.1. Pro-Health Antioxidant Activity of Rubus Fruits

Fruits of the genus *Rubus* are a rich source of bioactive chemical compounds, mainly antioxidants, including phenolic compounds. Several thousand polyphenolic molecules have been identified in the edible organs of various plants [1]. Secondary metabolites contained in *R. idaeus* fruits are represented by a number of antioxidants, e.g., flavonoids, vitamins, anthocyanins, terpenes, lipids, and amino acids, along with their derivatives, all strengthening the immune system [2,3,4]. The antioxidant phenolic compounds and other antioxidants contained in *R. idaeus* extracts serve many pharmacological functions [3,5]. The fruits of various *Rubus* species and other berries consumed as part of the diet can be used for the prophylaxis and adjuvant therapy of many diseases, mainly oxidative stress-induced conditions [6,7]. *Rubus* fruit phytochemicals exhibit a wide range of pharmacological activities, e.g., anti-obesity [8], anti-Alzheimer [9], antibacterial [6], anticancer [10], hepatoprotective [11], hypoglycemic [12], hypolipidemic [13], neuroprotective [14], against obesity [15], and skincare [16] effects.

#### 1.1.1. Antidiabetic Properties

Oxidative stress may lead to the development of diabetes, metabolic syndrome, and obesity. In diabetic rats, ellagic acid decreases blood glucose levels and increases the levels of serum insulin, mitigates glucose intolerance, and stimulates insulin secretion [17]. High ellagic acid content, i.e., 207–244 mg kg^−1^ f.w., was detected in the *R. idaeus* fruits cv. ‘Autumn Bliss’, ‘Heritage’, ‘Rubi’, and ‘Zeva’, and 18 other common raspberry cultivars and *R. occidentalis* L. ‘Bristol’ contained 120–324 mg/100 g of this compound [18,19]. Oleanolic and ursolic acids contained in *R. chingii* fruits inhibit α-glucosidase and exert a beneficial effect on diabetes therapy. These acids can serve as therapeutic agents in the prophylaxis and treatment of metabolic diseases. Additionally, methanol extracts of *R. chingii* fruits inhibit protein tyrosine phosphatase 1B [20]. Polyphenols contained in *R. chingii* fruits alleviate hyperglycemia in mice with type 1 diabetes through the enhancement of the antioxidant reaction and inhibition of the expression of Atrogin-1 and Trim63 proteins. Polyphenols also protect pancreatic β cells from damage and stimulate insulin secretion and improve glucose tolerance through the regulation of glycogen metabolism and the inhibition of gluconeogenesis via activation of insulin signaling [17]. Procyanidins contained in *R. amabilis* fruits exert hypoglycemic effects, reduce the intracellular production of reactive oxygen species and MDA, and simultaneously increase SOD activity. Through the activation of PI3K/Akt/FoxO1 signaling, procyanidins protect cells against apoptosis. Therefore, they can be used as one of the components of antidiabetic herbal medicines. Three-month therapy with *R. occidentalis* extracts administered at a dose of 900 or 1800 mg/day regulates glycemia and has a positive effect on vascular inflammation markers and the function of pancreatic β cells in prediabetic patients [21].

#### 1.1.2. Anti-Obesity Properties

Obesity is a cause of many diseases and metabolic dysfunctions, which are part of the so-called metabolic syndrome. The prophylaxis and adjuvant therapies for obesity necessitate the use of appropriate phytochemicals, e.g., vitamins and antioxidants [22,23]. Ellagic acid inhibits the expression of the gene responsible for white adipocytes in rat adipose tissue. It has a positive effect on obesity-induced dyslipidemia and on *UCP1* gene expression in brown adipocytes [24]. Ellagic acid and ethanolic extracts from *R. coreanus* fruits exhibit hypocholesterolemic and anti-obesity effects and downregulate the expression of adipogenic and lipogenic genes in high-fat diets. *R. coreanus* extract suppresses lipid accumulation in white adipose tissue and induces thermogenesis in brown adipose tissue [25]. *R. idaeus* fruit extract reduces triglyceride and cholesterol levels, as well as oxidative stress and lipid metabolism, body weight, and body fat in hyperlipidemic mice. Additionally, it regulates the *PPARa* and *Hmgcr* genes, downregulates *Hmgcr* gene expression, and accelerates the conversion of triglycerides into fatty acids. It has a protective role against hypertriglyceridemia and mitigates lipid metabolism disorders [26]. Polysaccharide compounds from *R. chingii* fruits protect against palmitic acid-mediated lipotoxicity, a reduction in mitochondrial membrane potential, and a decrease in glutathione levels [27].

#### 1.1.3. Anticancer Properties

Oxidative stress is one of the main factors in the pathogenesis of neoplastic cancer [28,29]. One of the causes of cancer development is the overproduction of reactive oxygen species leading to oxidative stress, cell metabolism disorders, damage to DNA and other metabolites, and pathological processes [30,31]. Ellagitannins, mainly sanguiin H-6 and lambertianin C, present in *R. idaeus* fruits exhibit a chemopreventive effect. They change the morphology of the nucleus and induce the apoptosis of Caco-2 cells; hence [32], *R. idaeus* extracts exhibit anticancer activity against human hepatocellular carcinoma HepG2 and mouse L20B cell lines. A strong cytotoxic and cytostatic effect on tumor cells has been noted in both lines [33]. Volatile substances present in berry fruit extracts inhibit the proliferation of non-small-cell lung cancer A549 cells via the induction of apoptosis [10]. Raspberry fruits used in the diet exert a chemopreventive effect on colorectal cancer in patients with non-specific inflammatory bowel disease. This disease, which is a chronic immune-mediated disease associated with an increased risk of colon dysplasia and colorectal cancer, is the third and fourth leading cause of cancer-related death in the United States and worldwide, respectively. Inflammation-induced carcinogenesis is associated with oxidative stress, genome instability, immune effectors, cytokine dysregulation, and the NFκB signaling pathway. Mainly anthocyanins were found to be involved in chemopreventive activity [34]. The bioactivity of blackberry fruit extracts is mainly related to the content of phenolic compounds and ursane-type triterpenes. The extracts have anticancer activity against colon cancer cells HT-29 and T-84 and anti-inflammatory effects through the inhibition of the secretion of pro-inflammatory cytokines IL-8 [6].

### 1.2. Rationale behind the Study

These investigations were undertaken given the increase in the commercial production of the *R. idaeus* repeat-fruiting cultivars ‘Polka’, ‘Pokusa’, and ‘Polana’. The demand for these fruits suggests the need for comparative studies of bioactive chemical compounds contained in *Rubi fructus*, which is increasingly being recommended as a superfood with health-promoting properties. The literature does not provide comprehensive comparative analyses of the antioxidant activity, selected antioxidants, or other nutritionally important metabolites in the fruits of raspberry cultivars grown in southeastern Poland. This issue is of paramount importance for dietetics and the potential use of raspberries in the production of functional food mainly for their rich antioxidant and dietary fiber content. It is also important for molecular biology to elucidate the molecular basis of the activity of bioactive food ingredients that can enhance, weaken, or modify the physiological and metabolic functions of the organism. Additionally, they can be used in research strategies: genomics, proteomics, and metabolomics, the development of tools for the acquisition of knowledge of active phytochemicals used as food ingredients, and the elucidation of the molecular basis of the biological activity of these compounds.

The aim of this study was to compare the antioxidant activity, determine the total content of selected primary and secondary metabolites (proteins, lipids, total sugars, available sugars, dietary fiber, polyphenols, flavonoids, anthocyanins, and vitamin C), and analyze the qualitative and quantitative composition of amino acids and fatty acids in the fruits of the *Rubus idaeus* L. repeat-fruiting cultivars ‘Pokusa’, ‘Polana’, and ‘Polka’, which are commonly cultivated in Europe and have been listed in the National Register of Horticultural Plant Varieties.

## 2. Materials and Methods

### 2.1. Study Material

**Figure 1:**
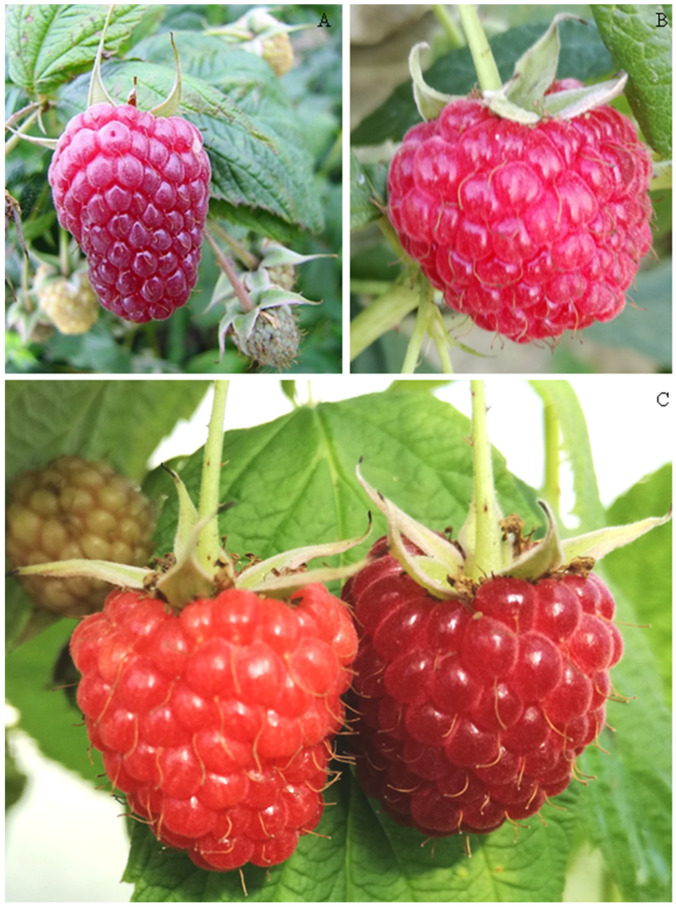
(**A**–**C**). *R. idaeus* ‘Pokusa’ (**A**), ‘Polana’ (**B**), and ‘Polka’ (**C**) fruits.

Fruits of three *Rubus idaeus* repeat-fruiting cultivars, ‘Polka’, ‘Polana’, and ‘Pokusa’ (Figure 1A–C), which are commonly cultivated in commercial production in Poland and Europe and have been listed in the National Register of Horticultural Plant Varieties, were collected in a commercial plantation located in Blinów II, Szastarka Commune, Lublin Province (50°52′53″ N 22°23′05″ E). Fertilization and plant protection agents were applied in accordance with the recommended raspberry cultivation calendar. The fruits of the three cultivars were harvested at the full maturity stage in September.

#### Origin and Lineage of the Analyzed Cultivars

Most commercial cultivars derive from *R. idaeus*. The Polish repeat-fruiting cultivars ‘Polana’, ‘Polka’, and ‘Pokusa’ are commonly grown in commercial production. ‘Polana’ is a cross between the American cultivar ‘Heritage’ and the English cultivar ‘Zeva Herbsternte’. It was entered into the National Register of Horticultural Plant Varieties on 13 February 1991 as the first repeat-fruiting raspberry cultivar. In turn, the cultivar ‘Polka’ was obtained by crossing the English cultivars ‘Autumn Bliss’ and ‘Lloyd George’ with *R. coreanus.* Tribes P 93563 and P 89141 are part of the lineage of this cultivar. ‘Polka’ was entered into the National Register of Horticultural Plant Varieties in 2003. Both *R. idaeus* cultivars have gained worldwide recognition and have contributed to the establishment of fruit quality standards. The lineage of ‘Pokusa’ includes two English cultivars: ‘Autumn Bliss’ and ‘Heritage’ and several species of the genus *Rubus: R. idaeus*, *R. strigosus*, *R. odoratus*, and *R. occidentalis* [35].

### 2.2. Determination of Antioxidant Activity

The antioxidant activity and total polyphenol content were determined in fresh raspberry fruit and juice. Samples of blended fruit and juice from the analyzed cultivars were subjected to extraction with the use of 100 mL of water at 100 °C. The fruit extract was filtered, and its appropriate amount was used in the subsequent analyses. The following methods were employed to determine the antioxidant potential: Folin–Ciocalteu (FC), ferric reducing antioxidant power (FRAP), and 2,2-diphenyl-1-picrylhydrazyl (DPPH). Total polyphenols were determined with the spectrophotometric method using the Folin–Ciocalteu (FC) reagent.

#### 2.2.1. FRAP Method (Ferric Reducing Antioxidant Power Assay)

The antioxidant activity of the *Rubi idaei fructus* raw material was assessed using the ferric reducing antioxidant power (FRAP) assay [36]. The standard curve was plotted for iron sulfate. The absorbance of the solution was measured at a wavelength of λ = 593 nm using a Hitachi U-2900 spectrophotometer (Hitachi, Tokyo, Japan). The absorbance values were converted into the FRAP unit, which expresses the amount of antioxidant compounds per 1 g of raw material (µmol/g) capable of reducing 1 mole of iron (III) to iron (II).

#### 2.2.2. DPPH Method

The antioxidant activity of the *Rubi fructus* raw material was determined as in Bondet et al. [37] with the use of the 2,2-diphenyl-1-picrylhydrazyl (DPPH) radical. A methanol solution of the DPPH radical (3.9 mL) with a concentration of 6 × 10^−5^ mol/L was added to a specified amount of the extract. After 30 min, a decrease in the absorbance of the solution was measured at a wavelength of λ = 515 nm. The absorbance result was converted into a Trolox unit based on the standard curve.

### 2.3. Chemical Analyses

The energy value and the content of selected bioactive compounds were determined in fresh *R. idaeus* fruits.

#### 2.3.1. Available Carbohydrates

The total sugar and available carbohydrate content in the analyzed fruits was determined with the Luff–Schoorl method [38]. The method is based on the reduction of Cu^2+^ ions contained in Luff’s solution by sugars present in the sample at pH = 9.5. The difference between the sodium thiosulfate (VI) utilized in the blank sample and in the reaction with copper (II) reduced by saccharides in the sample corresponds to the amount of copper reduced directly by the reducing sugars. 

#### 2.3.2. Total Fiber

The amount of fiber in the fruits was determined using the enzymatic method described by Asp et al. [39]. The sample was treated with α-amylase, pepsin, and pancreatin enzymes. The amount of undigested insoluble and soluble fiber precipitated from the filtrate was determined using the gravimetric method.

#### 2.3.3. Total Protein

The total nitrogen content in the fruit samples (*n* = 3) was determined with the Kjeldahl method [40] using a FOSS Kjeltec 2300 analyzer (Foss Tecator, Höganäs, Sweden). The total protein content was estimated following the Polish standard (PN-75 A-04018:2002) [41] by multiplying the nitrogen content by the protein factor: 5.6 [42].

#### 2.3.4. Amino Acids

The qualitative and quantitative composition of amino acids in the fruits was determined as in Davies and Thomas [43]. A 5 g aliquot was placed in an INGOS hydrolyzer thimble (INGOS, Prague, Czech Republic) and flooded with 6 M of HCl. The solution was saturated with nitrogen, hydrolyzed at 110 °C for 20 h, cooled, and filtered. The hydrolyzate was evaporated in an RVO 400 SD vacuum evaporator (Hamburg, Boeco, Germany) at 50 °C, washed with 1 mL of distilled water, and evaporated again. The dry residue remaining in the vacuum flask was dissolved in 5 mL of citrate buffer, pH 2.2. The sample was applied onto a 35 cm long column with a 5 mm diameter filled with ion exchange resin. Amino acids were separated using an AAA 400 amino acid analyzer (Ingos, Prague, Czech Republic) at temperatures of T1 = 60 °C and T2 = 63 °C. The amino acids were derivatized into colored amino acid–ninhydrin complexes and identified using a photometric detector at 570 nm and 440 nm for proline. The chromatogram was read in the Chromulan computer program.

#### 2.3.5. Total Fat Content

The total fat content was estimated in accordance with the Polish standard (PN-EN ISO 5508:1996) [44]. The Soxhlet method, based on the acid hydrolysis of 4 mol/dm^3^ HCl, was used in this analysis.

#### 2.3.6. Qualitative and Quantitative Composition of Fatty Acids

The quantitative and qualitative analysis of fatty acids was performed in accordance with the Polish standard (PN-EN ISO 12966-1:2014) [45]. The saponification process was carried out using a methanolic solution of potassium hydroxide. Next, esterification was performed by adding a methanolic boron trifluoride solution, and separation was carried out using hexane and a saturated sodium chloride solution. The hexane layer was collected into a glass vial and dried over anhydrous sodium sulfate. The chromatographic analysis was performed using a Varian 450-GC gas chromatograph (Temecula, CA, USA) equipped with an 1177 Split/Splitless injector at a temperature of 250 °C with a Select™ Biodiesel for FAME capillary column (30 m; 0.32 mm; 0.25 μm). The stationary phase included Select Biodiesel for FAME Fused Silica, a column furnace with a starting temperature of 100 °C and a final temperature of 240 °C, and a FID detector (temperature of 270 °C). The carrier gas (helium) flow rate was 1.5 mL/min. The Galaxie™ Chromatography Data System Autosampler Varian CP-8400 software (Version 3.3.5.) was used for the collection, integration, and calculation of results. (Version 1.9.302.530) from Bruker Daltonics (BrukerCorporation, Fremont, CA, USA)

#### 2.3.7. Folin–Ciocalteu Method

The total phenolic compound content was determined with the Folin–Ciocalteu method as in Prior [46]. Absorbance at λ = 765 nm was measured after 30 min with a Hitachi U-2900 spectrophotometer (Hitachi, Tokyo, Japan). The results were expressed in mg of phenolic compounds per 1 g of dry material as caffeic acid equivalents.

#### 2.3.8. Anthocyanins

The accumulation of anthocyanins in the fruits was determined using the modified Martínez and Favret method [47]. Anthocyanins were extracted via the maceration of fresh samples in a methanol:HCl solution (99:1, *v*/*v*) at 4 °C for 24 h. The extracts were centrifuged at 10,000× *g* rpm for 10 min at 4 °C, and their absorbance was read at 527 nm and 652 nm (Cecil CE 9500, Cecil Instruments, Cambridge, UK). The concentration of anthocyanins was calculated using the molar extinction coefficient (ε = 29,600 M−1 cm^−1^) for cyanidin 3-glycoside (C3G).

#### 2.3.9. Vitamin C

The vitamin C content was determined using HPLC according to the Polish standard (PN-EN 14130:2004) [48]. The extraction was carried out with the use of a metaphosphoric acid solution. In this process, dehydro-L(+) ascorbic acid is transformed into L(+) ascorbic acid. A 3 g fruit sample was treated with 80 mL of metaphosphoric acid and shaken; after filtration, a 100 mL flask was filled with the acid. To reduce the resulting solution (20 mL), 10 mL of an L-cysteine solution was added, trisodium phosphate was used to achieve pH = 7.0–7.2, and metaphosphoric acid was added. The vitamin C content was determined against the standard curve with the use of HPLC.

#### 2.3.10. Flavonoids

The total flavonoid content was determined with the method proposed by Lamaison and Carnet [49]. A raw material aliquot was transferred to a flask, and 20 mL of acetone, 2 mL of hydrochloric acid, and 1 mL of methenamine solution were added. The mixture was kept under reflux in a boiling water bath for 30 min. The hydrolyzate was filtered into a volumetric flask (100 mL) and supplemented with acetone. After transferring 20 mL of the solution into a separatory funnel, 20 mL of water was added, and the mixture was extracted with ethyl acetate in 15 mL portions and 3 times in 10 mL portions. The combined organic layers were washed with water twice (40 mL each), filtered into a volumetric flask (50 mL), and supplemented with ethyl acetate. The solution intended for the analyses was prepared by adding 2 mL of an aluminum chloride solution (20 g/L) to 10 mL of the stock solution followed by the addition of an acetic acid:methanol mixture (1:19) to a volume of 25 mL. Next, the reference solution was prepared by supplementing 10 mL of the stock solution with an acetic acid:methanol mixture (1:19) to 25 mL. After 45 min, the absorption of the solutions at 425 nm was measured and compared with the reference solution. The percentage of flavonoids was expressed as quercetin equivalents.

#### 2.3.11. Energy Value

The energy value of the analyzed fruits was determined according to EU Commission Delegated Regulation No. 78/2014 [50].

### 2.4. Statistical Analysis

The means of the measurements and the standard deviation were calculated. The significance of differences was analyzed statistically using the Statistica 6.0 software (StatSoft, Tulsa, OK, USA). Differences between selected traits were assessed with the use of one-way ANOVA. Statistical inference was performed at a significance level of *p* < 0.05.

## 3. Results

### 3.1. Selected Nutrients and Energy Value 

**Figure 2:**
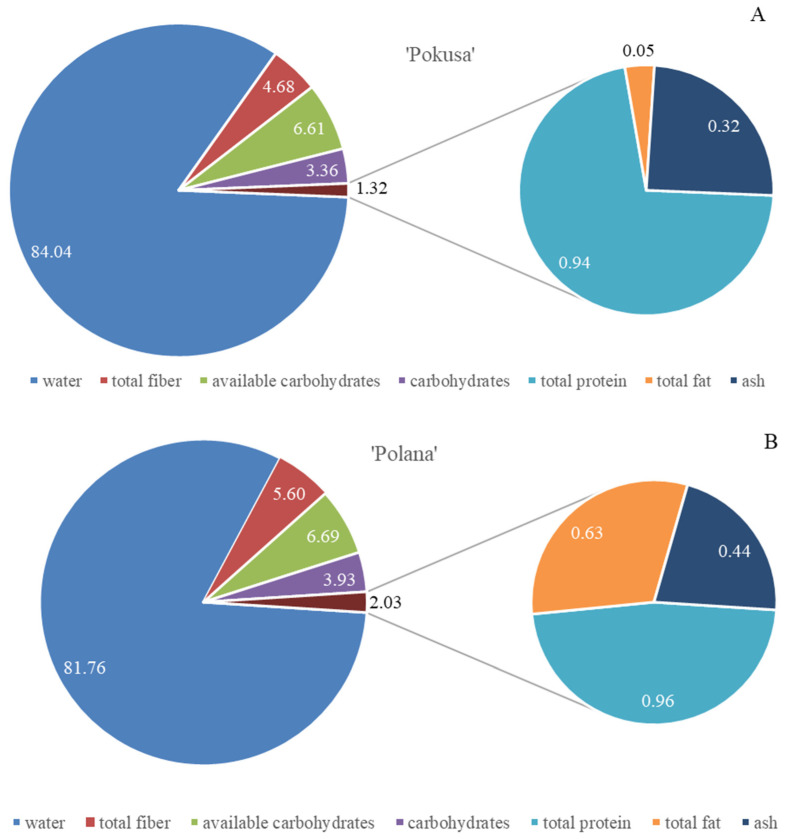

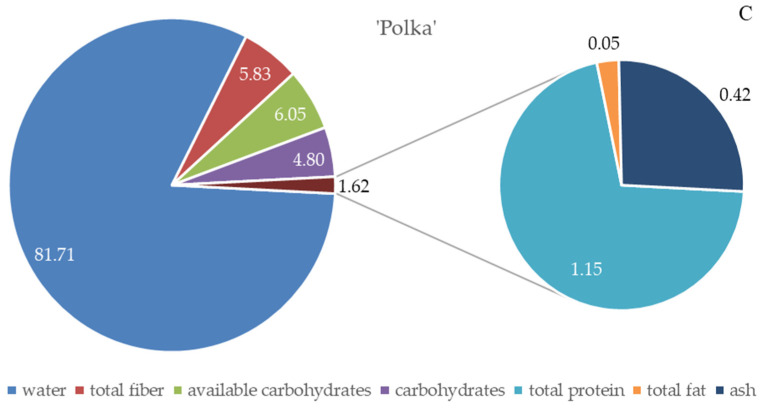
(**A**–**C**) Total water, protein, dietary fiber, sugar, available carbohydrate (% f.w.), fat, and ash (mg/100 g f.w.) content in the fruits of the repeat-fruiting *R. idaeus* cultivars: (**A**) ‘Pokusa’, (**B**) ‘Polana’, (**C**) ‘Polka’.

The total fiber content in the fruits of the analyzed ‘Pokusa’, ‘Polana’, and ‘Polka’ cultivars was 4.7, 5.6, and 5.8 g/100 g f.w., respectively. In turn, the content of total sugars and available carbohydrates ranged from 6.1 in ‘Polka’ to 6.7 g/100 g f.w. in ‘Polana’. The protein content ranged from 0.95 in ‘Pokusa’ and ‘Polana’ to 1.15 g/100 g f.w. in ‘Polka’. The amount of fat was estimated at approximately 0.05 mg/100 g f.w. in ‘Pokusa’ and ‘Polka’ and 0.63 mg/100 g f.w. in ‘Polana’. The levels of ash ranged from 0.32 (‘Pokusa’) to 0.42 mg/100 g f.w. (‘Polka’ and ‘Polana’). Therefore, the ‘Pokusa’ fruits had lower fiber and ash content than ‘Polka’ and ‘Polana’, but ‘Polka’ had a higher level of protein and lower amounts of available carbohydrates than ‘Pokusa’ and ‘Polana’ (Figure 2A–C).

**Figure 3:**
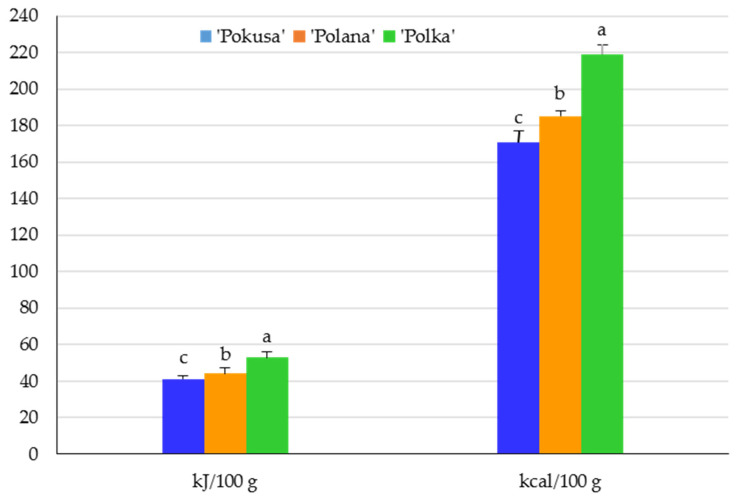
Energy value of the fruits of the repeat-fruiting *R. idaeus* cultivars. Notes: Means of the energy value expressed in kJ/100 g and in kcal/100 g marked with the same letter do not differ significantly between the cultivars at a significance level of α = 0.05.

The energy value of the fruits of the analyzed *R. idaeus* cultivars ranged from 171 in ‘Pokusa’ to 219 kJ/100 g in ‘Polka’. This parameter, converted into kcal/100 g, was in a range of 41–53. The value of this parameter in ‘Polana’ was significantly lower than in ‘Polka’ and markedly higher than in ‘Pokusa’ (Figure 3.)

### 3.2. Antioxidants: Vitamin C, Anthocyanins, and Flavonoids

**Figure 4:**
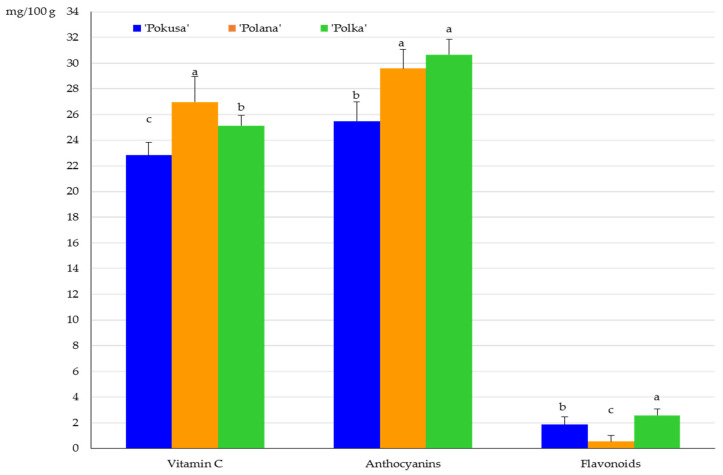
Total vitamin C, anthocyanin, and flavonoid content in the fruits of the repeat-fruiting *R. idaeus* cultivars. Notes: Means of vitamin C, anthocyanins, and total flavonoids marked with the same letter do not differ between the cultivars at a significance level of α = 0.05.

The total vitamin C content in the fruits of the *R. idaeus* cultivars was ranked as follows (mg/100 g): ‘Polana’ (27) < ‘Polka’ (25.1) < ‘Pokusa’ (22.8). The differences between the cultivars were statistically confirmed. In turn, the concentration of anthocyanins in the fruit ranged from 25.5 in ‘Pokusa’ to 30.7 mg/100 g in ‘Polana’. The value of this parameter in ‘Polana’ and ‘Polka’ did not differ significantly and was markedly higher than in ‘Pokusa’. The flavonoid contents in *Rubi fructus* from the ‘Polana’, ‘Pokusa’, and ‘Polka’ cultivars were 0.5; 1.9, and 2.6 mg/100 g, respectively. The value of this parameter in ‘Pokusa’ was significantly lower than in ‘Polka’ but higher than in ‘Polana’ (Figure 4).

The polyphenolic compound content in the analyzed fruits was determined with the Folin–Ciocalteu method. The concentration of polyphenols in Rubi fructus was similar in the three cultivars and ranged from 1.65 in ‘Polka’ to 1.7 mg/100 g in ‘Pokusa’ (Table 1). In turn, the fresh juice from the fruits of the three raspberry cultivars ‘Polana’, Pokusa’, and ‘Polka’ had substantially higher phenolic compound contents, i.e., 224, 251, and 260 mg/100 mL, respectively. The value of this parameter in ‘Polana’ was significantly higher than in ‘Pokusa’ and lower than in ‘Polka’ (Table 1).

### 3.3. Protein Amino Acids

**Figure 5:**
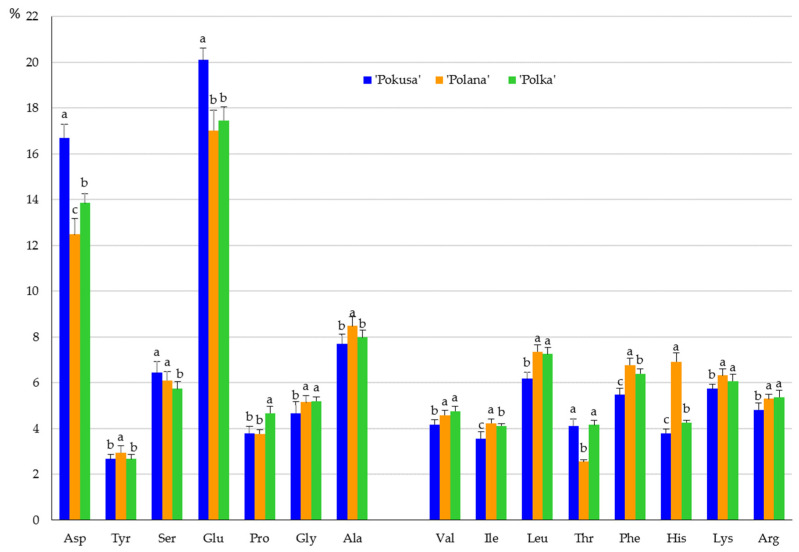
Amino acid content in the fruits of the repeat-fruiting *R. idaeus* cultivars. Notes: Means of each amino acid marked with the same letter do not differ between the cultivars at a significance level of α = 0.05.

The fruits of the analyzed cultivars contained 15 protein amino acids. In the total pool, the content of exogenous amino acids ranged from 56% in ‘Polana’ to 62% in ‘Pokusa’. They were dominated by leucine (from 6.2 in ‘Pokusa’ to 7.4% in ‘Polana’), phenylalanine (from 5.5 in ‘Pokusa’ to 6.8 in ‘Polana’), and lysine (from 5.7 in ‘Pokusa’ to 6.3% in ‘Polana). In the group of endogenous amino acids, the highest content was exhibited by glutamic acid (from 17 in ‘Polana’ to 20.1% in ‘Pokusa’), aspartic acid (from 12.5 in ‘Polana’ to 16.7% in ‘Pokusa’), and alanine (from 7.7 in ‘Pokusa’ to 8.5% in ‘Polana’) (Figure 5).

### 3.4. Fatty Acids 

**Figure 6:**
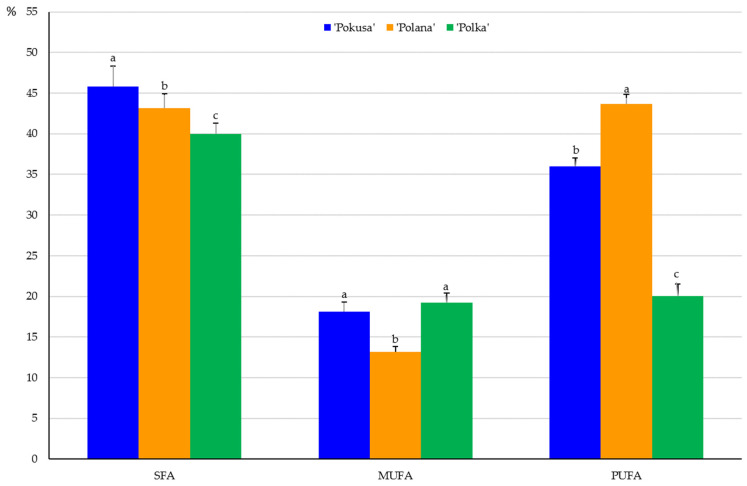
Percentage of saturated fatty acids (SFAs), monounsaturated acids (MUFAs), and polyunsaturated fatty acids (PUFAs) in the fruits of the repeat-fruiting *R. idaeus* cultivars. Notes: Means marked with the same letter for SFA, MUFA, and PUFA do not differ between the cultivars at a significance level of α = 0.05.

In the fruits of the analyzed cultivars, the content of saturated fatty acids (SFAs) in the total fatty acid pool ranged from 40% in ‘Polka’ to 45.8% in ‘Pokusa’. The SFA content in ‘Polana’ was significantly lower than in ‘Pokusa’ and markedly higher than in ‘Polka’. The levels of monounsaturated (MUFA) and polyunsaturated (PUFA) fatty acids were in a range from 13.2 in ‘Polana’ to 19.2% in ‘Polka’ and from 20.1 in ‘Polka’ to 43.7% in ‘Polana’, respectively (Figure 6). The MUFA content in ‘Pokusa’ and ‘Polka’ did not differ significantly and was markedly higher than in ‘Polana’. In turn, the PUFA content in ‘Pokusa’ markedly exceeded the value of this parameter determined in ‘Polka’ but was lower than in ‘Polana’.

**Figure 7:**
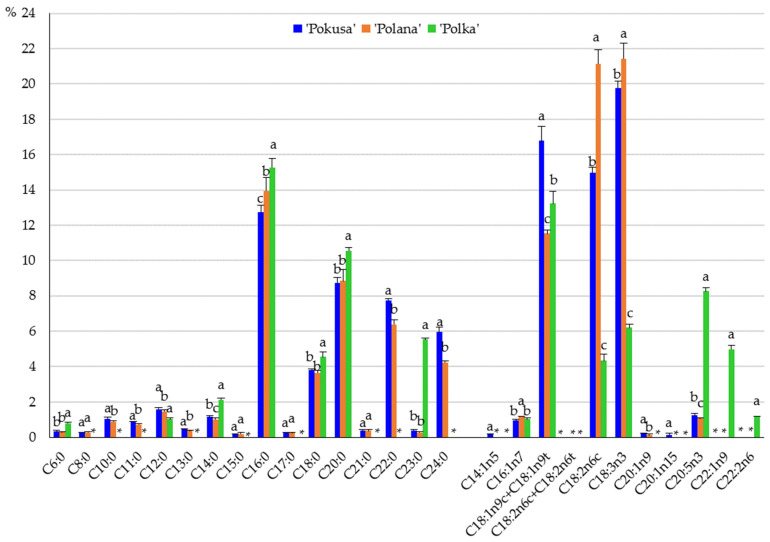
Content of saturated and unsaturated fatty acids in the fruits of the repeat-fruiting *R. idaeus* cultivars: ‘Pokusa’, ‘Polana’, ‘Polka’. Notes: C6:0 (caproic acid), C8:0 (caprylic acid), C10:0 (capric acid), C11:0 (undecanoid acid), C12:0 (lauric acid), C13:0 (tridecanoid acid), C14:0 (myristic acid), C15:0 (pentadecanoic acid), C16:0 (palmitic acid), C17:0 (margaric acid), C18:0 (stearic acid), C20:0 (arachidic acid), C21:0 (heneicosanoic acid), C22:0 (docosanoic acid, behenic acid), C23:0 (tricosanoic acid), C24:0 (tetracosanoic acid, lignoceric acid), C14:1n5 (9-tetradecenoic acid, myristoleic acid), C16:1n-7 (palmitoleic acid), C18:1n9c (oleic acid), C18:1n9t (elaidic acid), C18:3n6 gamma (gamma-linoleic acid), C18:3n3 (alpha-linolenic acid), C20:1n9 (11-eicosenoic acid), C20:1n15 (cis-5-eicosenoic acid), C20:5n3 (eicosapentaenoic acid), C22:2n-6 (docosadienoic acid). Means of a single fatty acid included in Figure 6 followed by the same letter do not differ between the cultivars at a significance level of α = 0.05. *—not detected.

In the monounsaturated fatty acid group, the sum of the oleic and elaidic acids dominated in the three cultivars and accounted for 16.8% (‘Pokusa’), 11.5% (‘Polana’), and 13.2% (‘Polka’). Palmitic acid in the cultivars ‘Pokusa’ and ‘Polana’ and erucic acid in ‘Polka’ were the second most abundant acids. The most abundant polyunsaturated acids were α-linolenic acid (19.8%) in ‘Pokusa’, linoleic acid (21.5%) in ‘Polana’, and eicosapentaenoic acid (8.3%) in ‘Polka’, as well as linoleic acid (15%) in ‘Pokusa’ and α-linolenic acid in ‘Polana’ (21.4%) and ‘Polka’ (6.2%). In turn, the most abundant saturated fatty acids in the fruits of the three cultivars were palmitic acid ranging from 12.7% in ‘Pokusa’ to 14% ‘Polana’; arachidic acid in ‘Pokusa’ (8.7%) and ‘Polana (8.8%); and tricosanoic acid in ‘Polka’ (5.5%) (Figure 7).

**Figure 8:**
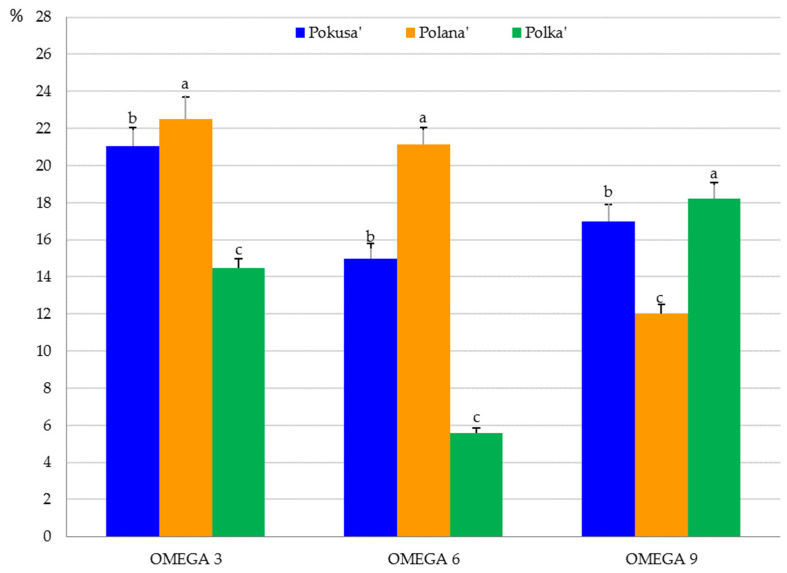
Omega-3, omega-6, and omega-9 fatty acid content in the fruits of the repeat-fruiting R. idaeus cultivars. Notes: Means of each omega fatty acid group (omega-3, omega-6, and omega-9) marked with the same letter do not differ between the cultivars at a significance level of α = 0.05.

Omega-3 acids dominated the fruits of the analyzed cultivars. The total content of these acids ranged from 14.5% in ‘Polka’ to 22.5% in ‘Polana’. This group was represented by linolelaidic, α-linolenic, eicosapentaenoic, and cis-13,16-docosadienoic acids. The omega-6 fatty acid content in the raspberry fruits ranged from 5.6% in ‘Polka’ to 21.2% in ‘Polana’. Linoleic acid was one of the fatty acids from this group. In turn, the omega-9 fatty acid content ranged from 12% in ‘Polana’ to 18.2% in ‘Polka’. This group of acids was represented by erucic, oleic, and elaidic acids. The raspberry fruits also contained palmitoleic acid from the omega-7 group (Figure 7 and Figure 8). The omega-3 and omega-6 fatty acid content in the ‘Pokusa’ fruits was substantially higher than in ‘Polka’ but lower than in ‘Polana’. In turn, the level of omega-9 fatty acid in ‘Pokusa’ was markedly higher than in ‘Polana’ and lower than in ‘Polka’ (Figure 8).

### 3.5. Antioxidant Activity

#### 3.5.1. Fruits

Using the FRAP method, the highest value of the iron ion reduction capacity of the fresh fruits was exhibited by ‘Polana’ (20.27 μmol/g), followed by ‘Pokusa’ (12.58 μmol/g) and ‘Polka’ (12.79 μmol/g), and the values of this parameter in ‘Polana’ were significantly higher than in the other two cultivars. The results of the DPPH radical reduction assay calculated into Trolox equivalents revealed that the fruits of the three raspberry cultivars had comparable antioxidant activity. The shortest time required for 50% DPPH radical reduction (T_EC50_ [s]) and indicating the highest antioxidant activity was the same in all three cultivars, i.e., 600 s.

**Figure 9:**
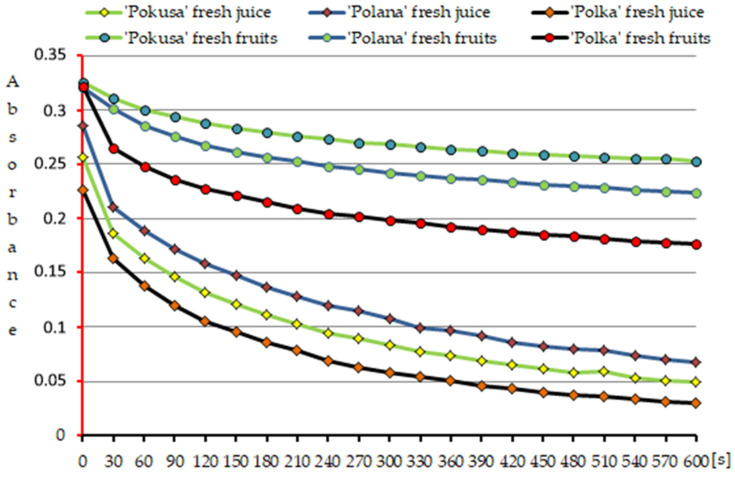
Kinetics of the color reaction of DPPH• radical neutralization by polyphenols contained in the fresh fruits and in the fresh juice from the fruits of the repeat-fruiting *Rubus idaeus* cultivars.

Another parameter of the antioxidant capacity of the fresh fruits was the antioxidant efficiency (AE) factor. This coefficient ranged between 0.0014 (‘Pokusa’) and 0.0019 dm^3^/µmol × s (‘Polka’). The value of this parameter in ‘Polana’ was significantly higher than in ‘Pokusa’ but lower than in ‘Polka’ (Table 2, Figure 9).

Additionally, since the fruits of the analyzed cultivars contained a mixture of different concentrations of various polyphenols and their derivatives, the kinetics of the neutralization of the DPPH• radical by these bioactive chemical compounds was determined (Figure 9). 

#### 3.5.2. Fresh Juice

As shown by the FRAP analysis of the antioxidant activity of the chemical compounds contained in the fresh juice from the fruits of the analyzed cultivars, the highest iron (II)-to-iron (III) reducing power was exhibited by ‘Pokusa’, i.e., 12317 μmol/100 mL. The juice from the cultivar ‘Polana’ reduced iron ions at a level of 12276 μmol/100 mL, and the lowest antioxidant activity was exhibited by ‘Polka’ (11138 μmol/100 mL).

As determined using the DPPH method, ‘Polana’ (the cultivar with the lowest antioxidant activity) required the longest time for a 50% reduction in the DPPH radical, i.e., 291 s. ‘Polka’ exhibited the greatest antioxidant capacity, with an average reduction time of 141 s, which indicated the highest antioxidant activity. The AE coefficient reflecting the antioxidant efficacy had the highest and lowest values in ‘Polka’ (0.008071 dm^3^/µmol × s) and ‘Polana’ (0.003479 dm^3^/µmol × s), respectively (Table 3, Figure 9).

## 4. Discussion

### 4.1. Phenolic Compounds

The Folin–Ciocalteu method revealed that the phenolic compound contents in the fresh raspberry fruits and juice were 1.65–1.71 mg/g and 223.7–260.1 mg/100 mL, respectively, expressed as caffeic acid equivalents. Cervantes et al. [51] reported that fresh raspberry fruit contained 140 mg gallic acid equivalents/100 g f.w. The anti-inflammatory activity of these compounds is related to their ability to interfere with oxidative stress signaling and the inhibition of pro-inflammatory signal transduction [2,52]. Viuda-Martos et al. [53] showed that the bioavailability of phenolic compounds, including phenolic acids and flavonoids, is determined by, e.g., the type of dietary fiber.

### 4.2. Fiber

The total fiber content in the *R. idaeus* fruits (4.7–5.8 g/100 g f.w.) was similar to the value of this parameter in common raspberry fruits reported by Baenas et al. [54] (6.1 g/100 g f.w.) and Noratto et al. [55] (6.5%). Raspberry fruit fiber consists mainly of insoluble compounds, is rich in phenolic compounds and antioxidants, and has antioxidant properties. It is characterized by good swelling properties, water and fat retention, glucose diffusion, and prebiotic activity associated with polyphenols. Fiber fractions from raspberry fruits can serve as the functional and prebiotic ingredients of food products [54]. They also adsorb cholesterol and glucose; hence, they can be a component of supplements used for the treatment of obesity [56]. Dietary fiber exerts a negative effect on the bioavailability of polyphenols, and pectins substantially reduce the total content of bioavailable phenolic compounds and monomeric anthocyanins. The interactions between phenols and pectins should be considered in designing diets, as the inclusion of pectins in red raspberry purée has been found to reduce the number of bioavailable polyphenols [57]. 

### 4.3. Anthocyanins

The anthocyanin content in the fruits analyzed in the present study was in a range of 25.5–30.7 mg/100 g. These values were higher than the concentration of anthocyanins in *R. idaeus* ‘Polana’ (19.3 mg/100 g f.w.) reported in the literature [58] and lower than in the common raspberry cultivars ‘Pokusa’, ‘Polana’, and ‘Polka’ (75.5–83.5 mg/100 g) [19]. Anthocyanins have therapeutic properties. These compounds contained in wild blackberry extracts are used as part of therapies improving the survival of glioblastoma patients [59]. Anthocyanins in *R. coreanus* fruits, i.e., cyanidin 3-O-sambubioside, cyanidin 3-O-glucoside, cyanidin 3-O-xylosylrutinoside, and cyanidin 3-O-rutinoside, are responsible for 47–55% of the total antioxidant capacity of phenolic compounds. These compounds exert a neuroprotective effect on PC-12 neuronal cells exposed to oxidative stress [60]. Additionally, anthocyanin fractions from *R. eubatus* ‘Hull’ fruits reduce free radicals and oxidative damage in keratinocytes. They also increase the expression of antioxidant enzymes, thereby protecting keratinocytes from UV-mediated oxidative damage [61]. The prophylactic anticancer dose of anthocyanins is approx. 2400 mg/kg per day [62]. In red, yellow, black, hybrid, and BR (blackberry) fruits of many varieties from different *Rubus* species, the color is determined by the composition of flavonoids, including anthocyanins. Differences in the concentration of cyanidin-3-sambubioside in the anthocyanin pathway have been found between these fruits [63].

### 4.4. Flavonoids

In the present study, the flavonoid content was 1.9 mg RUE/g^−1^ in ‘Pokusa’ and 2.6 mg RUE/g^−1^ in ‘Polka’. The values of this parameter were similar to those in the cultivars ‘Tulameen’ (1.98 mg RUE/g^−1^) and ‘Polka’ (2.16 mg RUE/g^−1^) [64] and higher than their content in *R. idaeus* (4.4–5.0 mg RUE/g ^1^) [65,66]. Flavonoids isolated from *R. fairholmianus* fruits have analgesic properties and can be used in the phytotherapy of free-radical-induced diseases [67]. The analgesic properties of phenolic-rich fractions from *R. occidentalis* are associated with antinociceptive effects on postoperative pain and their ability to bind to α2-adrenergic, nicotinic, cholinergic, and opioid receptors [68]. *R. idaeus* extract, which is rich in phenolic compounds, e.g., flavonoids, relieves pain in knee osteoarthritis patients; hence, it may be a component of non-steroidal anti-inflammatory drugs [69]. He et al. [20] found that triterpenoids, flavonoids, alkaloids, and phenolic acids present in *R. chingii* are involved in antioxidant, anti-inflammatory, and anticancer effects. The anticancer activity of flavonoids consists of regulating enzymes involved in scavenging ROS, cell cycle arrest, the induction of apoptosis, and the inhibition of tumor cell proliferation and invasion. Flavonoids act as antioxidants in cancer cells, participate in apoptotic pathways, and downregulate pro-inflammatory signaling pathways [70]. Genes involved in flavonoid biosynthesis and genes associated with plant hormone signal transduction are two key elements of the adaptation of *Rubus corchorifolius* to the environment. Flavonoids protect plants against ultraviolet-light- and high-temperature-induced stresses [71]. Using the favorable *R. coreanus* traits for selection and breeding programs and the *R. occidentalis* genome for reference-guided assembly, Chen et al. [72] found that several biological pathways are disrupted and sixteen pathways are enriched during fruit development and ripening, with the involvement of genes responsible for flavonoid biosynthesis; phenylpropanoid biosynthesis; plant hormone signal transduction; and cutin, suberin, and wax biosynthesis.

### 4.5. Vitamin C

In the present study, the vitamin C content in the fresh fruits of the analyzed cultivars (mg/100 g), i.e., 22.8 in ‘Pokusa’; 25.13 in ‘Polka’; and 27.0 in ‘Polana, was similar to that in the fruits of 11 preselected wild-grown common raspberry genotypes and the cultivar ‘Heritage’ (21–36 mg/100 g) described in the literature, with the wild raspberry fruit samples having, in general, higher ascorbic acid content than the cultivated fruits [73]. Similar vitamin C content in fresh *R. idaeus* fruits (22.1–31.2 mg/100 g) was shown in a study conducted by de Ancos et al. [18], where the values of this parameter in the late cultivars ‘Zeva’ and ‘Rubi’ were higher than in the early cultivars ‘Autumn Bliss’ and ‘Heritage’. Kazimierczak et al. [74] reported higher vitamin C content in the cultivars ‘Polka’ (42.2–47.8 mg/100 g) and ‘Polana’ (34.6–43.6 mg/100 g) than the values obtained in the present study. In contrast to ‘Polka’, ‘Polana’ fruits from the conventional cultivation system exhibited a higher value of this parameter than those from the organic plantation. Ponder and Hallmann [75] reported that fruits from four raspberry cultivars of ‘Polka’ collected in autumn had 30.2 mg/100 g of this substance. The fruits of the conventionally grown raspberries contained significantly higher vitamin C content than organic raspberries, i.e., 40.5 and 33.7 mg/100 g, respectively. The ascorbic acid content of ‘Meeker’ raspberries was 20.1 mg/100 g. In ‘Heritage’, ‘Autumn Bliss’, and ‘Fallgold’ twice-bearing fruit, it was in a range of 31.0–32.4, 31.0–37.7, and 16.8–18.5 mg/100 g, and fruits collected in the summer contained higher levels of ascorbic acid than those harvested in autumn. The ascorbic acid content in red-black *Rubus fructicosus* fruits was between 14.3 and 17.5 mg/100 g. In turn, the red-colored raspberry × blackberry hybrid ‘Tayberry Sunberry’ and the black-colored ‘Silvan’ contained 19.7, 28.0, and 18.4 mg/100 g of ascorbic acid [76]. Akimov et al. [77] reported that the vitamin C content in 18 promising raspberry varieties was in the range of 13.6–31.1 mg/100 g. Surya et al. [78] found that ripe *R. fraxinifolius* fruits had high vitamin C content (82.97 mg/100 g), whereas a lower amount of this substance was found in *R. rosifolius* (54.30 mg/100 g).

Vitamin C plays a role in the prophylaxis of cardiovascular diseases, dilates and seals blood vessels, and regulates blood pressure and cholesterol levels, thereby reducing the risk of atherosclerosis and coronary heart disease. It supports the immune system through the stimulation of cell activity. In physiological conditions, ascorbic acid increases the absorption of calcium, iron, and copper and helps to alleviate allergic reactions. It serves as a co-factor and a proton donor in the activation of fundamental enzymes in the biosynthesis of collagen, steroid hormones, and L-carnitine and is involved in the amidation of some peptide hormones and the formation and metabolism of amino acids or hydroxyphenyl alanine [79].

Vitamin C is a powerful antioxidant involved in the first line of defense offering protection for lipid membranes and proteins. It inhibits the oxidation of carbohydrates and nucleic acids and is an excellent source of high-energy electrons for free radicals [80].

Vitamin C is an electron donor for ascorbate peroxidase involved in the glutathione-ascorbate cycle, which operates in the cytosol, mitochondria, chloroplast stroma, and peroxisomes. Vitamin C plays a role in plant responses to abiotic stresses, including osmotic stress, drought, chilling, heat, and heavy-metal toxicity. Vitamin C has been found to be responsible for 3% of the total antioxidant power of *R. idaeus* fruits [81]. It is estimated that the minimum daily requirement of vitamin C in adults is 40–60 mg. Both fresh and processed common raspberry fruits are an excellent source of vitamin C in the diet. The consumption of 100 g of fresh raspberry fruit covers the daily requirement for vitamin C in approximately 50% of female and 30% of male adolescents [79,82].

### 4.6. Amino Acids

In the fruits of the tested cultivars, exogenous amino acids accounted for 56–62% of the total pool, with a dominance of leucine, phenylalanine, and lysine. Leucine, i.e., the main component of the anabolic response, is a measure of protein quality in the nutrition of athletes. A 2–3 g leucine dose is sufficient to increase muscle protein synthesis [83]. As demonstrated in clinical studies, leucine regulates muscle protein synthesis and has an impact on phosphorylation via the mechanistic target of rapamycin complex 1 and protein–protein interaction, thus facilitating the anabolic process [84]. Additionally, a leucine-restricted diet alleviates obesity-related problems, improves insulin resistance, promotes white adipose tissue browning, upregulates the expression of neurotrophic factors, and inhibits neural inflammation in the brain region associated with memory functions. It also changes the intestinal microflora by reducing the number of inflammation-associated bacteria and increasing the number of short-chain fatty acids associated with the growth of some bacteria; consequently, it may alleviate some obesity-related problems [85].

### 4.7. Fatty Acids

The fruits of ‘Pokusa’ and ‘Polana’ were dominated by linoleic acid (15–21.5%), linolenic acid (19.8–21.4%), and palmitic acid (12.7–15.3%). These values were higher than in the common raspberry fruits (9.2%) analyzed by Zorzi et al. [86]. In turn, the linolenic and palmitic acid content, i.e., 27.2 and 17.9%, respectively, was lower than the values shown by these authors. Similarly, a study of fruits of 11 raspberry genotypes and one cultivar showed the dominance of linoleic acid (42.2–52.6%), linolenic acid (17.8–24.1%), and palmitic acid (4.9–9.1%) [87]. In the present study, SFAs accounted for 40–45.8% of the total pool of fatty acids contained in the fruits of the three *R. idaeus* cultivars. These values are similar to those determined by other authors in the fruits of *R. idaeus* ‘Kweli’ (41.7%) and the common raspberry (46%) [86,88]. The monounsaturated fatty acid content shown in the present study (13–19%) was similar to that of the fruits of six other *R. idaeus* cultivars (14.7–18.5%) [89] and lower than in common raspberry fruits (22.7%) [86] and *R. idaeus* ‘Kweli’ (29.2%) [89]. The polyunsaturated fatty acid content in the fruits of the analyzed cultivars (from 20% in ‘Polka’ to 44% in ‘Polana’) was lower than in the fruits of six other *R. idaeus* cultivars (62.9–78.7%) [89]. The PUFA content in ‘Pokusa’ was similar to the amount detected in common raspberry and *R. idaeus* ‘Kweli’ fruits (29.1–31.3%) [86,88]. The PUFA/SFA ratios in ‘Pokusa’, ‘Polana’, and ‘Polka’ calculated in the present study were 0.79, 1.01, and 2.0, respectively. In comparison with the ratio in *R. idaeus* ‘Kweli’ (0.7) [88], the present values were similar for ‘Pokusa’ but higher for ‘Polana’ and ‘Polka’. The concentrations of ω-3, ω-6, and ω-9 fatty acids in the fruits of ‘Pokusa’, ‘Polana’, and ‘Polka’ were in a range of 14.5–22.5, 5.6–21.2, and 12–18%, respectively. The ω-6/ω-3 ratios in the fruits were 0.7, 0.9, and 0.4, respectively. These results were lower than in *R. idaeus* ‘Kweli’ fruits (1.4) [88]. PUFA/SFA and ω-6/ω-3 PUFA ratios are important indicators of the pro-health quality and nutritional value of food products. Their values (>0.45 and <4) evidence the high nutritional quality and health-promoting properties of the raspberries described as nutritional products in the present study and in other literature reports [86,88].

### 4.8. Antioxidant Activity

In the present study, the iron ion reduction capacity (FRAP method) of the fresh fruits and juice ranged from 10.3 in ‘Polana’ to 12.8 μmol/g in ‘Polka’ and from 11138 in ‘Polka’ to 12317 μmol/100 mL in ‘Pokusa’. Marić et al. [5] reported that the antioxidant activity of *R. idaeus* seed extract (FRAP) was in a range of 72–118 µmol Fe^2+^/g. Cekiç and Özgen [90] showed a level of iron ion reduction of 11.2–19.8 µmol Fe^2+^/g in their study of wild and cultivated *R. idaeus* fruits. The kinetics of DPPH radical reduction via the antioxidant compounds contained in *R. idaeus* fresh fruits and juice was determined using three parameters; the first one showed the percentages of remaining unreduced radicals (DPPH rem %), which were estimated at 83.2–86.6 and 142–292, respectively. The second parameter indicated the time (s) required to reduce the initial radical concentration by 50% (T_EC50_) (600 s—fruits; 142–292 s—juice). The third parameter, i.e., the antioxidant efficacy (AE), was 0.0014–0.0019 dm^3^/µmol × s and 0.004–0.008 dm^3^/µmol × s, respectively. Gramza-Michałowska et al. [91] determined the same parameters in five berry fruit species, *Fragaria ananasa*, *Punica granatum*, *Rubus fruticosus*, *Rubus ideaus*, and *Vaccinium macrocarpon*, and found that the common raspberry and cranberry exhibited moderate antioxidant activity. Gülçin et al. [92] performed DPPH, ABTS^+^, DMPD^+^, and O_2_^−^ assays and found that lyophilized aqueous fruit extracts from domesticated and wild ecotypes of raspberries were effective antioxidants and free radical scavengers. This indicated that the phenolic-rich extract was a readily available source of natural antioxidants. *R. chingii* raspberry fruit extract inhibits the expression of TGF-β1 antibodies and reduces the expression of p-Smad2/3 signaling molecules and Smad4 protein. Concurrently, it attenuates TGF-β1 signal transduction and reduces the activation and proliferation of hepatic stellate cells (HSCs), thereby alleviating liver fibrosis in mice [93]. Martins et al. [94] investigated the health-promoting properties of *Rubus fruticosus* and *R. ulmifolius* and found that reactive species are products of normal cellular metabolism. Biologically active compounds, mainly phenolic compounds, are involved in signal transduction, growth regulation, gene expression, and immune response pathways. Exogenous antioxidants, e.g., flavonoids, stilbenes, and tannins, scavenge and detoxify radical oxygen species and prevent their generation through the modulation of signal transduction and their impact on metabolic processes, cell cycle, and apoptosis.

### 4.9. Applications

With their low calorific value and total sugar content, favorable profiles of amino acids and fatty acids, high concentrations of total fiber and antioxidants (polyphenols, flavonoids, anthocyanins, and vitamins), and free radical scavenging potential, the fruits of the *R. idaeus* ‘Pokusa’, ‘Polana’, and ‘Polka’ cultivars can be used in many industries. Given their antioxidant potential, PUFA indices, and ω-3 and ω-6 content, the analyzed *Rubi fructus* can serve as functional food products, superfoods, and nutraceuticals, as their polyphenolic compounds, flavonoids, anthocyanins, and vitamins contribute to free-radical scavenging and the inhibition of oxidative stress. Hence, the consumption of raspberry fruits should be recommended and encouraged. This suggests that researchers, dieticians, and pharmaceutical and cosmetic industries should develop strategies to use this raw material in the prophylaxis and adjuvant therapies of many diseases, as raspberry fruits have an appropriate nutritional and chemical profile and antioxidant compounds mitigating the harmful effects of oxidative stress.

### 4.10. Future Research

There is a need for further studies of many other biennial fruiting and repeat-fruiting cultivars commonly grown in commercial production, as well as wild genotypes, in order to analyze the pro-health metabolites involved in the total antioxidant capacity of this raw material and to elucidate the mechanisms of metabolic pathways and relevant interrelations between certain bioactive chemical compounds. An important role in metabolism is played by appropriate amino acid profiles regulating the nutritional value of *Rubi idaei fructus*. The present results can serve as a theoretical basis for the improvement of the quality of *Rubus* fruits and their more frequent use in the food, pharmaceutical, and cosmetic industries. Future research should be focused on a detailed analysis of natural anthocyanin pigments, which can be used as one of the main natural coloring ingredients, and on the safety of their use in food, pharmaceutical, and biocosmetological products. Another issue to be addressed in future studies is the utilization of fruit processing waste material in accordance with sustainable production and consumption principles, e.g., oil extraction from raspberry seeds. The knowledge of primary and secondary metabolites can help to determine fruit quality and prompt the choice of appropriate raspberry cultivars with high nutritional value enhanced by the flavonoid, polyphenolic compound, anthocyanin, and vitamin content. A precisely determined metabolome may provide additional information about similarities and differences in the chemical profile of primary and secondary metabolites between cultivars. It will also help to assess fruit quality and apply genetic engineering for the development of new raspberry cultivars with the best genomes to encode desired traits. Additionally, an important aspect is the use of wild raspberry genotypes, which are an important source of health-promoting metabolites and have high nutritional metabolite contents, to develop new varieties with a high concentration of phytochemicals responsible for the antioxidant capacity of raspberry fruits. Clinical trials should be conducted to determine the safety of use, tolerance, and intake doses in agreement with the prophylaxis and therapy requirements of specific diseases. Future studies are also expected to provide important data on the mechanisms of the metabolic pathways of free-radical scavenging and oxidative stress inhibition via bioactive antioxidants. Another area of research may be focused on the assessment of the expression of genes regulating enzyme activity, anthocyanin biosynthesis, and pigment metabolism with the use of structural genes and regulatory transcription factors.

## 5. Conclusions

Given their low calorific value, available carbohydrate and total sugar content, and glycemic index, as well as high dietary fiber, vitamin C, anthocyanin, nutritional unsaturated fatty acid, and important exogenous amino acid content, fruits of repeat-fruiting *R. idaeus* cultivars can be a valuable additive to the human diet and a component of superfoods recommended for pro-health and slimming diets. In the fruits of the examined cultivars, exogenous amino acids account for 56–62%, with a dominance of leucine, phenylalanine, and lysine. The endogenous amino acid group was dominated by glutamic acid, aspartic acid, and alanine. Unsaturated fatty acids represented 35–60%, and the MUFA group was dominated by oleic, elaidic, palmitic, and erucic acids. The acids of this group are known to prevent oxidative modifications of lipoproteins. In turn, α-linolenic, eicosapentaenoic, and linoleic acids were the most abundant PUFAs, and palmitic, arachidic, and tricosanoic acids dominated in the SFA group. MUFAs from the ω-9 group accounted for 12–18%, and the levels of ω-3 and ω-6 PUFAs were estimated at 15–23 and 6–21%, respectively. It is recommended that the daily dietary intake of these compounds should be below 10% of total energy. These acids supplement nutrients and regulate cholesterol and glucose metabolism. The content of polyphenolic compounds in the analyzed fruits was similar. The following order of the total polyphenolic content was established in the fresh fruit juice from the analyzed cultivars: ‘Pokusa’ < ‘Polana’ < ‘Polka’. The different antioxidant capacity assays used in the study confirmed the high antioxidant power of the fruits and fresh juice from *R. idaeus* ‘Pokusa’, ‘Polana’, and ‘Polka’ and indicated the potential use of *Rubi fructus* as a ‘fruit life’ ingredient of healthy food, supplements, and pharmaceutical and cosmetic products. The antioxidant activity of many raw materials of various species is associated with the presence of, e.g., phenolic compounds, vitamins, anthocyanins, and carotenoids and depends on the cultivar, species, and soil and environmental factors.

## Figures and Tables

**Table 1:** Total phenolic compound contents in the fresh fruits and fresh juice of the repeat-fruiting *R. idaeus* cultivars.

Cultivar	Folin–Ciocalteu			
	Min.–Max.	Mean ± SD	Min.–Max.	Mean ± SD
	Fruits (mg/g f.w.)		Juice (mg/mL f.w.)	
‘Pokusa’	1.69–1.73	1.71 ± 0.02 ^a^	216.45–227.36	223.72 ± 6.30 ^c^
‘Polana’	1.59–1.75	1.68 ± 0.09 ^a^	232.81–260.70	250.98 ± 15.7 ^b^
‘Polka’	1.57–1.73	1.65 ± 0.08 ^a^	243.71–276.42	260.07 ± 16.3 ^a^

Explanations: Fruit/juice means for the phenolic compound content parameters followed by the same letter do not differ between the cultivars at a significance level of α = 0.05.

**Table 2:** Antioxidant activity of fresh fruits of the repeat-fruiting *Rubus idaeus* cultivars.

Cultivar	FRAP μmol/g		Kinetics of DPPH• Radical Reduction				
			T_EC50_ [s]	DPPH rem %		AE dm³/µmol × s	
	Min.–Max.	Mean ± SD	Mean ± SD	Min.–Max.	Mean ± SD	Min.–Max.	Mean ± SD
‘Pokusa’	12.51–12.64	12.59 ± 0.07 ^b^	600 ± 0.0	86.17–87.23	86.61 ± 0.55 ^a^	0.00140–0.00143	0.00142 ± 0.00002 ^c^
‘Polana’	9.09–11.60	20.27 ± 1.26 ^a^	600 ± 0.0	82.71–86.97	85.20 ± 2.21 ^a^	0.00159–0.00163	0.00161 ± 0.00002 ^b^
‘Polka’	12.28–13.17	12.79 ± 0.46 ^b^	600 ± 0.0	78.72–92.29	83.24 ± 7.83 ^a^	0.00189–0.00197	0.00193 ± 0.00004 ^a^

Notes: For the fruits, means of antioxidant activity parameters followed by the same letter do not differ between the cultivars at a significance level of α = 0.05.

**Table 3:** Antioxidant activity of the fresh juice of the repeat-fruiting *Rubus idaeus* cultivars.

Cultivar	FRAP μmol/g		Kinetics of DPPH• Radical Reduction					
			T_EC50_ [s]		DPPH rem %		AE dm³/µmol × s	
	Min.–Max.	Mean ± SD	Mean ± SD	Min.–Max.	Mean ± SD	Min.–Max.	Min.–Max.	Mean ± SD
‘Pokusa’	10000.01–13780.49	12317.08 ± 2029.6 ^a^	245–298	276.67 ± 27.97 ^b^	77.66–78.99	78.28 ± 0.67 ^a^	0.00328–0.00385	0.00352 ± 0.0003 ^b^
‘Polana’	12012.20–12743.90	12276.42 ± 405.9 ^b^	234–352	291.67 ± 59.05 ^a^	72.61–77.93	76.06 ± 3.00 ^b^	0.00286–0.00422	0.00348 ± 0.0006 ^c^
‘Polka’	10000.01–12378.05	11707.32 ± 948.5 ^c^	120–182	141.67 ± 34.96 ^c^	59.84–60.37	60.20 ± 0.31 ^c^	0.00630–0.00907	0.00807 ± 0.0015 ^a^

Notes: For the juice, means of antioxidant activity parameters followed by the same letter do not differ between the cultivars at a significance level of α = 0.05.

## Data Availability

The data presented in this study are available in this article.
